# Reduction in the incidence of myocardial infarction in patients with rheumatoid arthritis who respond to anti–tumor necrosis factor α therapy: Results from the British Society for Rheumatology Biologics Register

**DOI:** 10.1002/art.22809

**Published:** 2007-09

**Authors:** W G Dixon, K D Watson, M Lunt, K L Hyrich, A J Silman, D P M Symmons, British Society for Rheumatology Biologics Register Control Centre Consortium

**Affiliations:** University of ManchesterManchester, UK

## Abstract

**Objective:**

Rheumatoid arthritis (RA) is associated with an increased risk of coronary artery disease, possibly acting via shared mechanisms of inflammation. This study was undertaken to test the hypothesis that the powerful antiinflammatory effect of anti–tumor necrosis α (anti-TNFα) therapy might lead to a reduction in the incidence of myocardial infarction (MI) in patients with RA.

**Methods:**

Using data from the British Society for Rheumatology Biologics Register, a national prospective observational study, we compared MI rates in 8,670 patients with RA treated with anti-TNFα and 2,170 patients with active RA treated with traditional disease-modifying antirheumatic drugs (DMARDs).

**Results:**

Through July 2006, 63 MIs occurred in the anti-TNFα cohort during 13,233 person-years of followup and 17 MIs occurred in the DMARD cohort during 2,893 person-years of followup, equivalent to a rate of 4.8 events per 1,000 person-years and 5.9 events per 1,000 person-years, respectively. After adjustment for baseline risk factors, there was no reduction in the rate of MI in the anti-TNFα cohort compared with the DMARD cohort (incidence rate ratio 1.44 [95% confidence interval 0.56–3.67]). In an analysis of anti-TNFα–treated patients who responded to the treatment within 6 months versus those who did not, MI rates were found to be 3.5 events per 1,000 person-years in responders and 9.4 events per 1,000 person-years in nonresponders. The adjusted incidence rate ratio (95% confidence interval) for responders compared with nonresponders was 0.36 (0.19–0.69).

**Conclusion:**

These results indicate that RA patients treated with anti-TNFα do not have a lower incidence of MI compared with RA patients treated with traditional DMARDs. However, the risk of MI is markedly reduced in those who respond to anti-TNFα therapy by 6 months compared with nonresponders. This finding supports the notion that inflammation plays a pivotal role in MI.

It is now well established that rheumatoid arthritis (RA) is associated with increased mortality and morbidity due to accelerated atherosclerosis, including from myocardial infarction (MI) (1–5). This increased risk cannot be attributed to traditional cardiovascular risk factors, such as smoking and hypertension, alone (2,5,6). There is mounting evidence that the increased risk is related to the overall burden of inflammatory disease in RA (7,8). In addition, atherosclerosis itself is increasingly being viewed as an inflammatory condition (9).

The cytokine tumor necrosis factor α (TNFα) plays a key role in the pathogenesis of RA (10). Introduction of the anti-TNFα therapies infliximab, etanercept, and adalimumab has dramatically improved the outcome of severe RA beyond that achieved with traditional disease-modifying antirheumatic drugs (DMARDs) (11–13). Proinflammatory cytokines, including TNFα, are involved in modification of lipid profile and insulin resistance (14) and the initiation and progression of atherosclerosis (15,16), hemostasis (17), and atherosclerotic plaque rupture, the most common event leading to an acute MI (15). Inhibition of TNFα in patients with RA may therefore lead to a reduction in MI rates by inhibiting one or more of these mechanisms. However, some patients do not respond well to anti-TNFα drugs. Therefore, we hypothesized that any reduction in the incidence of MI would be limited to those patients who displayed a good clinical response to TNFα.

The aims of this study were, first, to determine whether the incidence of MI in RA patients treated with anti-TNFα was lower than that in patients treated with traditional DMARDs and, second, to explore the impact of response to treatment on the rates of MI in the anti-TNFα cohort. To date, 1 study has shown a reduced rate of all cardiovascular events following anti-TNFα therapy (18), but no published studies have so far explored MI incidence or the influence of treatment response.

## PATIENTS AND METHODS

### Patients

Subjects were participants in a large national prospective observational study, the British Society for Rheumatology Biologics Register (BSRBR). Methods of patient recruitment and followup have been described in detail elsewhere (19). Briefly, the study aims to recruit all UK patients with rheumatic diseases treated with biologic agents, and an appropriate comparison group, in order to examine the long-term safety of these drugs. UK national guidelines recommend that anti-TNFα drugs be reserved for patients with active RA, defined as a Disease Activity Score in 28 joints (DAS28) (20) >5.1 despite previous therapy with at least 2 DMARDs, one of which should be methotrexate (21), and that “any clinician prescribing these medications must (with the patient's permission) undertake to register the patient with the [BSRBR] and forward information on dosage, outcome and toxicity on a six-monthly basis” (22).

#### Anti-TNFα cohort

This cohort was restricted to patients registered with the BSRBR who were diagnosed as having RA and were treated with an anti-TNFα drug. Patients who were registered with the BSRBR >6 months after the start of biologic therapy were excluded. Patients had to have been followed up for ≥6 months by July 31, 2006.

#### Comparison cohort

A cohort of patients with active RA who have never taken biologic agents is being recruited in parallel with the anti-TNFα cohort by the BSRBR Control Centre Consortium (see Appendix A for a list of BSRBR Control Centre Consortium members). The comparison cohort is followed up using the same methodology used to follow up the anti-TNFα cohort (19). The patients in the comparison cohort were diagnosed as having active RA (guideline DAS28 ≥4.2) despite current treatment with a traditional DMARD. These patients also had to have been followed up for ≥6 months by July 31, 2006.

### Baseline assessment

Baseline data assessed in both cohorts included demographic characteristics, disease duration, 28-joint counts for swelling and tenderness, erythrocyte sedimentation rate and/or C-reactive protein level, and patient global assessment, and enabled calculation of a DAS28 score (20). Details of all previous and current DMARD therapy and all other current medications were obtained. Patients completed a Health Assessment Questionnaire (HAQ) adapted for British use (23). Townsend scores of multiple deprivation were calculated based on the patient's area of residence and compared with UK quintiles (24). Data were also collected on other variables that might influence cardiovascular risk, including all baseline drugs, body mass index (BMI), prior cardiovascular comorbidity (including previous MI, angina, and hypertension), diabetes, and smoking history.

### Followup

Data on changes in therapy, disease activity, and the occurrence of adverse events were collected in 3 ways. Rheumatologists were sent a questionnaire every 6 months, patients were sent a 6-month diary in which to document all hospital admissions, new medications, and new hospital referrals, and all patients were flagged with the UK General Register Office, which provides the BSRBR with information on deaths and cause of death (coded according to the International Statistical Classification of Diseases and Related Health Problems, Tenth Revision) (25). If an MI was reported from any of the 3 sources, further supporting information, such as a hospital discharge summary, was requested from the rheumatologist.

All available clinical information on the MIs was reviewed by 2 physicians (WGD and KLH) independently to verify the diagnosis according to an adapted European Society of Cardiology (ESC)/American College of Cardiology (ACC) definition (26). ESC/ACC criteria define an acute, evolving, or recent MI as a typical rise and fall of biochemical markers of myocardial necrosis with ≥1 of the following: ischemic symptoms, Q waves, ischemic electrocardiography changes, coronary artery intervention, or pathologic findings of an acute MI. Additional BSRBR verification criteria were thrombolysis and/or MI recorded on death certificate, regardless of autopsy confirmation. Any disagreement was resolved by consensus following discussion. Only verified MIs were included in the analysis.

### Statistical analysis

Response to treatment in the anti-TNFα cohort was defined according to European League Against Rheumatism criteria (27). Responders were those patients who achieved either a good or a moderate response, i.e., a reduction in the DAS28 score from baseline to 6 months of >1.2, or a reduction of >0.6 in addition to a DAS28 score of ≤5.1 at 6 months. DAS28 scores were not measured at 6 months in the DMARD cohort, and thus response could not be assessed in this group.

For patients in the anti-TNFα cohort, person-years of followup included only the time during which they were actively treated with the first anti-TNFα drug. The date of drug discontinuation was defined as the date of the first missed dose. Person-years were calculated from the first day of anti-TNFα therapy to the date of the most recent completed followup form prior to July 31, 2006, drug discontinuation, first MI, or death, whichever came first. Thus, only the first MI for an individual patient was analyzed. For the purposes of the current analysis, only MIs occurring while the patient was undergoing active treatment were attributable to that drug. If an MI occurred after a patient discontinued the first anti-TNFα agent, that event was not included in the analysis, regardless of whether the patient had been started on a new anti-TNFα drug.

For patients in the comparison cohort, person-years of followup included the time from the date of registration until the date of the most recent completed followup form prior to July 31, 2006, first MI, or death, whichever came first. For patients initially registered in the comparison cohort who subsequently received an anti-TNFα drug, person-years of followup in the comparison cohort included the time up to the date the anti-TNFα drug was started, and person-years of followup in the anti-TNFα cohort included the subsequent time, during which they were actively treated with the anti-TNFα drug.

Incidence rates of MI are presented as events per 1,000 person-years, with 95% confidence intervals (95% CIs). Incidence rate ratios were calculated using Poisson regression, initially comparing the anti-TNFα cohort with the DMARD cohort, and then comparing responders with nonresponders within the anti-TNFα cohort. Stepwise adjustment was performed, first for age and sex, and then additionally for RA disease severity (using baseline DAS28 score, HAQ, and disease duration as continuous variables), BMI, social deprivation (Townsend quintiles), cardiovascular comorbidity (previous MI, angina, hypertension), diabetes, smoking status (current, ever, or never), and baseline use of selected drugs (corticosteroids, nonsteroidal antiinflammatory drugs, lipid-lowering drugs, and antiplatelet drugs). All analyses were performed using Stata, version 8.2 software (StataCorp, College Station, TX).

## RESULTS

A total of 10,755 patients were included in the analysis: 8,659 in the anti-TNFα cohort (3,844 receiving etanercept, 2,944 receiving infliximab, and 1,871 receiving adalimumab) and 2,170 in the comparison cohort. Seventy-four patients switched from the comparison cohort to the anti-TNFα cohort and were included in both cohorts. Baseline characteristics are shown in Table 1. The comparison cohort included proportionally more men, and patients in this cohort were older than those in the anti-TNFα group. As anticipated, patients in the comparison cohort had less severe disease of shorter duration and had a lower rate of steroid use at baseline. There were significantly higher rates of previous MI and angina in the DMARD cohort, and significantly higher rates of use of lipid-lowering and antiplatelet drugs. Proportionally more patients in the DMARD cohort were in the most socially deprived quintile. As mentioned above, these differences were adjusted for in subsequent analyses.

**1:** Baseline characteristics of the DMARD-treated and anti-TNFα–treated patients*

Characteristic	DMARD (n = 2,170)	All anti-TNFα (n = 8,659)	Anti-TNFα nonresponders (n = 1,638)	Anti-TNFα responders (n = 5,877)	*P*†
Age, mean ± SD years	60 ± 12	56 ± 12‡	57 ± 12	56 ± 12	0.002
Sex, % female	72	76‡	79	76	0.01
DAS28 score, mean ± SD	5.0 ± 1.4	6.6 ± 1.0‡	6.4 ± 1.1	6.6 ± 1.0	<0.001
HAQ score, mean ± SD	1.5 ± 0.8	2.1 ± 0.6‡	2.2 ± 0.5	2.0 ± 0.6	<0.001
Disease duration, median (IQR) years	7 (1–15)	12 (6–19)‡	11 (6–19)	11 (6–19)	0.64
BMI, mean ± SD kg/m^2^	26.9 ± 5.7	26.7 ± 5.8§	26.9 ± 6.2	26.7 ± 5.7	0.27
Smoking history, no. (%)¶					0.07#
Current smoker	537 (25)	1,886 (22)	382 (23)	1,231 (21)	
Former smoker	849 (39)	3,298 (38)	625 (38)	2,241 (38)	
Never smoked	767 (35)	3,431 (40)	621 (38)	2,369 (40)	
Prior MI, no. (%)	116 (5.3)	250 (2.9)‡	48 (2.9)	154 (2.6)	0.48
Angina, no. (%)	183 (8.4)	381 (4.4)‡	85 (5.2)	240 (4.1)	0.04
Hypertension, no. (%)	672 (31.0)	2,581 (29.8)	506 (30.9)	1,731 (29.4)	0.19
Diabetes, no. (%)	132 (6.1)	470 (5.4)	110 (6.7)	287 (4.9)	0.003
Corticosteroids, no. (%)	418 (19.3)	3,793 (43.7)‡	743 (45.3)	2,519 (42.9)	0.06
Lipid-lowering drugs, no. (%)	338 (15.6)	768 (8.9)‡	159 (9.7)	502 (8.5)	0.14
Antiplatelet drugs, no. (%)	291 (13.4)	648 (7.5)‡	130 (7.9)	418 (7.1)	0.26
NSAIDs, no. (%)	1,344 (61.9)	5,705 (65.9)**	995 (60.8)	3,961 (67.4)	<0.001
Townsend quintile, no. (%)¶,††					0.001#
1	314 (14.5)	1,388 (16.0)	260 (15.9)	973 (16.6)	
2	301 (13.9)	1,469 (17.0)	250 (15.3)	1,021 (17.4)	
3	382 (17.6)	1,633 (18.9)	279 (17.0)	1,154 (19.6)	
4	443 (20.4)	1,881 (21.7)	380 (23.2)	1,263 (21.5)	
5	655 (30.2)	1,933 (22.3)	402 (24.6)	1,217 (20.7)	

* The number of anti–tumor necrosis factor α (anti-TNFα) responders and the number of anti-TNFα nonresponders do not equal the total number of anti-TNFα–treated patients, since information on the change in Disease Activity Score in 28 joints (DAS28) from 0 to 6 months was not available in 1,153 patients. For smoking history, prior myocardial infarction (MI), angina, hypertension, diabetes, corticosteroids, lipid-lowering drugs, antiplatelet drugs, nonsteroidal antiinflammatory drugs (NSAIDs), and Townsend quintiles, data were not available in all patients. HAQ = Health Assessment Questionnaire; IQR = interquartile range; BMI = body mass index.

† Anti-TNFα nonresponders versus anti-TNFα responders.

‡ *P* < 0.001 versus disease-modifying antirheumatic drug (DMARD)–treated patients.

§ *P* = 0.04 versus DMARD-treated patients.

¶ *P* for trend < 0.001.

# *P* for trend.

** *P* = 0.001 versus DMARD-treated patients.

†† Quintile 1 represents the least socially deprived; quintile 5 represents the most socially deprived.

The median followup was 1.66 years in the anti-TNFα cohort and 1.34 years in the DMARD cohort. There were 69 reported MIs in the anti-TNFα cohort compared with 20 in the comparison cohort. Of these, 63 and 17, respectively, were verified (Table 2). The crude incidence rate of MI was lower in the anti-TNFα cohort (4.8 events per 1,000 person-years) than in the DMARD cohort (5.9 events per 1,000 person-years), equivalent to an incidence rate ratio (95% CI) of 0.81 (0.47–1.38). However, after adjustment for age and sex, the incidence rate ratio increased to 1.13, and further adjustment for RA disease severity, social deprivation, traditional cardiovascular risk factors, and relevant baseline drug use resulted in an incidence rate ratio (95% CI) of 1.44 (0.56–3.67). Inclusion of the nonverified MIs in the analysis resulted in an adjusted incidence rate ratio (95% CI) of 0.97 (0.42–2.22). When analysis was limited to patients who had no prior MI or angina, the adjusted incidence rate ratio (95% CI) for verified MIs was largely unchanged at 1.38 (0.46–4.18). Rates of MI were higher in men, as expected, in both cohorts. There was again no significant difference in risk of MI with anti-TNFα therapy for either sex.

**2:** Incidence rates of verified first MI in DMARD-treated and anti-TNFα–treated patients*

	All patients		Male patients		Female patients	
			
	DMARD (n = 2,170)	Anti-TNFα (n = 8,659)	DMARD (n = 615)	Anti-TNFα (n = 2,072)	DMARD (n = 1,555)	Anti-TNFα (n = 6,587)
Person-years	2,893	13,233	831	3,199	2,062	10,034
No. of reported MIs	17	63	10	27	7	36
Rate of MIs per 1,000 person-years (95% CI)	5.9 (3.4–9.4)	4.8 (3.7–6.1)	12.0 (5.8–22.1)	8.4 (5.5–12.2)	3.4 (1.4–7.0)	3.6 (2.5–5.0)
Incidence rate ratio	Referent	0.81 (0.47–1.38)	Referent	0.70 (0.34–1.45)	Referent	1.06 (0.47–2.37)
Incidence rate ratio, adjusted for age and sex	Referent	1.13 (0.65–1.96)	Referent	0.92 (0.43–0.98)	Referent	1.39 (0.62–3.14)
Incidence rate ratio, multivariate analysis†	Referent	1.44 (0.56–3.67)	Referent	0.96 (0.26–3.55)	Referent	2.07 (0.62–6.88)

* 95% CI = 95% confidence interval (see Table 1 for other definitions).

† Adjusted for age, sex, disease severity, body mass index, social deprivation, smoking history, comorbidity, and baseline drug use.

Information on change in DAS28 score from 0 to 6 months was not available for 1,144 of the anti-TNFα–treated patients. In the remaining 7,515 patients, the incidence of MI in responders to anti-TNFα treatment was 3.5 events per 1,000 person-years, compared with 9.4 events per 1,000 person-years in nonresponders, with an adjusted incidence rate ratio (95% CI) of 0.36 (0.19–0.69) (Table 3). This effect was slightly stronger in men (adjusted incidence rate ratio 0.31 for men and 0.46 for women). Inclusion of the nonverified MIs resulted in an adjusted incidence rate ratio (95% CI) of 0.43 (0.23–0.80).

**3:** Incidence rates of verified first MI in nonresponders and responders to anti-TNFα treatment*

	Nonresponders (n = 1,638)	Responders (n = 5,877)
Person-years	1,815	9,886
No. of reported MIs	17	35
Rate of MIs per 1,000 person-years (95% CI)	9.4 (5.5–15.0)	3.5 (2.5–4.9)
Incidence rate ratio	Referent	0.38 (0.21–0.67)
Incidence rate ratio, adjusted for age and sex	Referent	0.38 (0.22–0.68)
Incidence rate ratio, multivariate analysis†	Referent	0.36 (0.19–0.69)
Incidence rate ratio by sex, multivariate analysis†		
Male patients	Referent	0.31 (0.12–0.81)
Female patients	Referent	0.46 (0.20–1.06)

* 95% CI = 95% confidence interval (see Table 1 for other definitions).

† Adjusted for age, sex, disease severity, body mass index, social deprivation, smoking history, comorbidity, and baseline drug use.

Followup rates in the BSRBR were excellent. Only 2.2% of all patients who were registered >12 months prior to July 31, 2006 had no returned rheumatologist questionnaires and 17.2% had no returned patient diaries. Only 0.6% had no followup information from either source. Patients with no returned followup information from the rheumatologist were not included in this analysis.

## DISCUSSION

We have shown that there is no overall reduction in MI rate in the short term in anti-TNFα–treated RA patients, compared with DMARD-treated patients with active RA who had never taken biologic agents. However, in RA patients who responded to anti-TNFα therapy, the risk of MI was reduced by more than half compared with nonresponders. This is consistent with the hypothesis that suppression of inflammation may reduce cardiovascular risk. Because we did not measure DAS28 scores in the DMARD cohort at 6 months, we cannot determine whether reduction in RA disease activity secondary to any treatment is associated with a reduction in MI incidence, or whether this finding is specific to anti-TNFα therapy.

This early reduction in MI risk in responders cannot be extrapolated to indicate a long-term beneficial cardiac effect. Raised levels of TNFα in chronic heart failure (28), along with the predictive value of high levels of TNFα on adverse outcome in chronic heart failure (29), led to studies to explore the potential benefit of anti-TNFα therapy in severe chronic heart failure. Contrary to expectations, 2 studies of etanercept had to be terminated early when interim analysis showed a lack of efficacy (30), and high-dose infliximab was shown to be detrimental in patients with moderate to severe chronic heart failure (31).

After MI, TNFα inhibition may potentially have both harmful and beneficial effects on myocardial function. TNFα has a protective role in the physiologic adaptive response to injury and limits infarct size (32), although when overexpressed it can lead to maladaptive effects, such as promoting left ventricular dysfunction (33). Evidence of the effect of anti-TNFα therapy on heart failure in RA patients without preexisting heart failure is currently very limited.

Despite the pleiotropic effects of TNFα inhibition on the heart, the focus of the current investigation was solely on MI rates during this early period of exposure. Given the link between plaque inflammation and plaque rupture (34), it is plausible that there might be an early reduction in MI incidence following anti-TNFα treatment, while potential effects on heart failure, if any, may only be noticeable over a longer time period.

There are important methodologic issues which must be considered when interpreting these data. The BSRBR is an observational study aimed at nationwide ascertainment of data related to anti-TNFα treatment, using an appropriate comparison group. As anticipated, patients in the anti-TNFα cohort had more severe disease. This difference should, if anything, have placed the anti-TNFα cohort at increased risk of MI. Conversely, the comparison cohort had higher rates of baseline cardiovascular risk factors, with greater baseline prevalence of ischemic heart disease, a higher percentage of current smokers, and a higher rate of self-reported lipid-lowering and antiplatelet drug use. We adjusted for these differences in the analysis. Nonetheless, better definition of cardiovascular risk, such as knowledge of actual cholesterol levels, may have attenuated the observed protection in our responder group.

Although most traditional cardiovascular risk factors were captured, exercise was not measured in the present study. Responders would be better able to exercise than nonresponders, lowering their cardiovascular risk. However, such a difference would only be apparent following their response to treatment and is not likely to have a large bearing on MI rates within this short followup period.

Anti-TNFα–treated patients were categorized according to the change in DAS28 score from baseline to 6 months. These data were missing at 1 or both time points in 1,153 patients (∼15%), and we therefore explored whether this missing data led to any bias. The MI rate in the patients without a categorized response was 7.7 events per 1,000 person-years. This rate is approximately halfway between the observed rates in the responder and nonresponder groups, suggesting that these data may be missing at random.

The BSRBR has a number of strengths. The size of the cohort far exceeds that of any anti-TNFα clinical trial. The aim is to recruit all RA patients in the UK treated with these agents and thus represent real-life practice. It is a condition of prescribing these agents in the UK National Health Service that the rheumatologist register the patient with the BSRBR (22). Although we cannot accurately assess the completeness of registration, estimates from various sources have suggested a capture rate of ≥70%.

It is important to remember that the risk of MI is increased in patients with RA independent of the treatment they receive. The inclusion of a cohort of DMARD-treated patients who had never taken biologic agents enabled us to estimate the risk reduction attributable to anti-TNFα drugs, which would not be possible if MI rates were compared with those in the general population. By using 3 sources of identification of MIs, we increased the chances of identifying all adverse events. Prior to verification, there were 20 MIs reported in the DMARD cohort and 69 in the anti-TNFα cohort. Following collection of available clinical information, 59 (10 in the DMARD cohort and 49 in the anti-TNFα cohort) were verified according to the ESC/ACC criteria. A further 21 (7 in the DMARD cohort and 14 in the anti-TNFα cohort) had either a death certificate diagnosis of MI or received thrombolysis. There was insufficient clinical information available on the remainder (3 in the DMARD cohort and 6 in the anti-TNFα cohort) to allow verification. A secondary analysis including these few nonverified MIs did not noticeably change the results.

It is also appropriate to determine whether the observed MI rate in the comparison group was similar to the expected outcome, which would enhance the external validity of the result. When compared with published data on MI rates in the general population (35) and the risk conferred by RA (5,36), the observed rates in the DMARD cohort of 12.0 and 3.4 events per 1,000 person-years for men and women, respectively, suggest that there was no major under- or overascertainment. Our findings are also consistent with those of a recent study from Sweden (18), which examined the effect of anti-TNFα therapy on all cardiovascular events combined and showed a nonsignificant decrease in the incidence of first-time severe cardiovascular events. Low numbers of events (n = 13) and a short followup time (656 person-years) in the anti-TNFα cohort in that study precluded further analysis (18).

This study showed no protective effect against MI in RA patients treated with anti-TNFα therapy compared with patients treated with traditional DMARDs, after adjustment for baseline risk. However, the suppression of joint disease with anti-TNFα therapy in RA patients may be associated with an early reduced risk of MI. This finding supports the notion that inflammation plays a pivotal role in the pathophysiology of MI.

## AUTHOR CONTRIBUTIONS

Dr. Symmons had full access to all of the data in the study and takes responsibility for the integrity of the data and the accuracy of the data analysis.

**Study design.** Dixon, Watson, Hyrich, Silman, Symmons.

**Acquisition of data.** Dixon, Watson, Hyrich, Symmons.

**Analysis and interpretation of data.** Dixon, Lunt, Hyrich, Silman, Symmons.

**Manuscript preparation.** Dixon, Lunt, Hyrich, Silman, Symmons.

**Statistical analysis.** Dixon, Lunt.

## ROLE OF THE STUDY SPONSOR

The BSRBR was established primarily to investigate the safety of biologic agents in routine practice. The financial support to the BSRBR comes indirectly from the following UK companies marketing biologic agents in the UK: Schering-Plough, Wyeth Laboratories, Abbott Laboratories, and Amgen, but the independence of the BSRBR and its investigators is assured in the following manner. The resources used to fund the BSRBR are received under contract by the BSR, which then provides a research grant under a separate contract to the University of Manchester, allowing the investigators normal academic freedom in relation to the data, their analysis, and use. Under the terms of the contract between the BSR and the sponsoring pharmaceutical companies, all publications are sent in advance to the companies prior to submission, for the purposes of information. The companies can, if they wish, point out factual errors. Any comments are vetted by 3 members of the steering committee, who decide whether they should be passed on to the authors. All publications are also reviewed by the BSR, but the material presented and the views expressed in all publications from the BSRBR are those of the authors and do not necessarily represent the views of the BSR.

## APPENDIX A: BSRBR CONTROL CENTRE CONSORTIUM

The BSRBR Control Centre Consortium consists of the following institutions (all in the UK): Antrim Area Hospital, Antrim (Nicola Maiden); Cannock Chase Hospital, Cannock Chase (Tom Price); Christchurch Hospital, Christchurch (Neil Hopkinson); Derbyshire Royal Infirmary, Derby (Sheila O'Reilly); Dewsbury and District Hospital, Dewsbury (Lesley Hordon); Freeman Hospital, Newcastle-upon-Tyne (Ian Griffiths); Gartnavel General Hospital, Glasgow (Duncan Porter); Glasgow Royal Infirmary, Glasgow (Hilary Capell); Haywood Hospital, Stoke-on-Trent (Andy Hassell); Hope Hospital, Salford (Romela Benitha); King's College Hospital, London (Ernest Choy); Kings Mill Centre, Sutton-In-Ashfield (David Walsh); Leeds General Infirmary, Leeds (Paul Emery); Macclesfield District General Hospital, Macclesfield (Susan Knight); Manchester Royal Infirmary, Manchester (Ian Bruce); Musgrave Park Hospital, Belfast (Allister Taggart); Norfolk and Norwich University Hospital, Norwich (David Scott); Poole General Hospital, Poole (Paul Thompson); Queen Alexandra Hospital, Portsmouth (Fiona McCrae); Royal Glamorgan Hospital, Glamorgan (Rhian Goodfellow); Russells Hall Hospital, Dudley (George Kitas); Selly Oak Hospital, Selly Oak (Ronald Jubb); St. Helens Hospital, St. Helens (Rikki Abernethy); and Withington Hospital, Manchester (Paul Sanders).
